# A featural account for own-face processing? Looking for support from face inversion, composite face, and part-whole tasks

**DOI:** 10.1177/20416695221111409

**Published:** 2022-07-06

**Authors:** Jasmine K. W. Lee, Steve M. J. Janssen, Alejandro J. Estudillo

**Affiliations:** School of Psychology, 69861 University of Nottingham Malaysia, Semenyih, Selangor, Malaysia; School of Psychology, 69861 University of Nottingham Malaysia, Semenyih, Selangor, Malaysia; School of Psychology, 69861 University of Nottingham Malaysia, Semenyih, Selangor, Malaysia;; Department of Psychology, 6657 Bournemouth University, Poole, Dorset, UK

**Keywords:** face perception, holistic processing, featural processing, self-face processing

## Abstract

It is widely accepted that face perception relies on holistic processing. However, this holistic advantage is not always found in the processing of the own face. Our study aimed to explore the role of holistic and featural processing in the identification of the own face, using three standard, but largely independent measures of holistic face processing: the face inversion task, the composite face task, and the part-whole task. Participants were asked to identify their face, a friend’s face, and an unfamiliar face in three different experimental blocks: (a) inverted versus upright; (b) top and bottom halves of the face aligned versus misaligned; and (c) facial features presented in isolation versus whole foil face context. Inverting a face impaired its identification, regardless of the identity. However, alignment effects were only found when identifying a friend or an unfamiliar face. In addition, a stronger feature advantage (i.e., better recognition for isolated features compared to in a whole-face context) was observed for the own face compared to the friend and unfamiliar faces. Altogether, these findings suggest that the own face is processed in a more featural manner but also relies on holistic processing. This work also highlights the importance of taking into consideration that different holistic processing paradigms could tap different forms of holistic processing.

Faces are important and salient social stimuli for humans. Human faces belong to a rather homogenous category with a similar basic arrangement of features: a pair of eyes aligned horizontally above a central nose and a mouth. Despite little variance among the underlying structure of faces, individuals are able to demonstrate remarkable expertise in the recognition of familiar faces. For instance, an image of a face can vary in expression, hairstyle, or luminosity, yet individuals are able to show the recognition of familiar faces with high accuracy and good reliability (Maurer et al., 2002). Furthermore, studies have also proposed that 250 ms is sufficient to identify a familiar face (e.g., Grill-Spector & Kanwisher, 2005; Jacques & Rossion, 2006). Given the accuracy and speed when identifying familiar faces, Carey (1992) suggested that most individuals are qualified as “face experts” for familiar faces (Young & Burton, 2018).

It has been classically assumed that face perception relies on holistic processing (Gauthier et al., 2003; McKone, 2009; Rossion, 2013). Although definitions of holistic processing vary (McKone, 2009; Rossion, 2008), holistic face processing is generally described as a strong perceptual integration of facial features rather than the perception of isolated facial features (for reviews, see McKone & Yovel, 2009; Rossion, 2013). Three experimental paradigms have been widely considered as the standard measures of holistic face processing: the face inversion task (Yin, 1969), the composite face task (Young et al., 1985), and the part-whole task (Tanaka & Farah, 1993; for a review, see Piepers & Robbins, 2012; Tanaka & Gordon, 2011).

Inverting a face impairs face identification, such that longer response times and lower accuracy are observed when identifying inverted faces compared to upright faces (i.e., face inversion effect [FIE]; Yin, 1969). As upright faces are generally processed holistically, face-specific processes are claimed to be disrupted or suppressed when a face is turned upside down (Rossion, 2008). To compensate for this disruption, inverted faces are processed in a more featural manner instead (Barton et al., 2006). Notably, this FIE is significantly larger and evident for face stimuli compared to other visual object stimuli (Yin, 1969). Furthermore, the finding that inverting face stimuli is particularly disruptive for familiar faces seems to suggest the importance of holistic processing for familiar faces. For example, with a familiarity judgement task, Caharel et al. (2006) found that participants were slower to respond to inverted familiar faces but not to inverted unfamiliar faces. Mirroring these findings, Keyes and Brady (2010) also showed that individuals tended to recognize friends’ faces faster than unfamiliar faces in upright but not in inverted conditions, postulating that holistic processing of faces underlies such a finding.

The composite face effect (CFE) shows that two identical top parts of a face are identified as different if they are aligned with different bottom parts (Rossion, 2013; Young et al., 1985). The composite effect was interpreted to demonstrate that individuals automatically perceive two halves as a “whole” (i.e., integrating two face halves) when they are aligned to form an upright face template (Susilo et al., 2013). Critically, by misaligning the top and bottom halves, the information for the “whole” face is disrupted, eliminating the effect (Young et al., 1985). Whereas the composite effect is generally not observed for inverted faces and object stimuli (e.g., Macchi Cassia et al., 2009; Rossion, 2008), composite effects are reported to be present for objects of expertise (Wong et al., 2009). Nevertheless, the observed effects for objects of expertise are typically smaller compared to those reported for faces (DeGutis et al., 2012).

Holistic face processing has also been demonstrated with the part-whole task. This task shows that facial features are identified better when presented in the context of the whole face than when presented alone (Tanaka & Farah, 1993). The part-whole effect (PWE) seems to show that facial features are not encoded as isolated units but that they are encoded and integrated as one “whole” perceptual unit (an upright face; Tanaka & Farah, 1993). Despite empirical evidence showing a PWE for recognizing objects (i.e., “object superiority effect”; Davidoff & Donnelly, 1990), the effect is, however, not reported for all objects (e.g., houses), and the PWE is substantially larger and consistently reported for faces (Tanaka & Farah, 1993).

Although these tasks are considered to measure the same processes, recent evidence seems to suggest that the face inversion, composite face, and PWEs tap into different notions of holistic processing (Rezlescu et al., 2017; Richler et al., 2012). Specifically, Rezlescu et al. (2017) tested the relations between the performances on these three tasks. Their findings showed that these measures correlated poorly with each other, indicating that these tasks might tap distinct perceptual mechanisms. In other words, the mechanisms behind the face inversion, composite face, and part-whole tasks seem to reflect different forms of holistic processing.

## Own-Face Processing

One's own face holds a significant meaning to humans as it is strongly tied to one's identity and one's self-consciousness (e.g., Estudillo & Bindemann, 2017a) and the ability to recognize one's own face helps to maintain a sense of self (Estudillo & Bindemann, 2017b; Platek et al., 2004). Being a significant stimulus critical to one's identity and the most relevant face to each individual, the own face seems to be processed in a quantitatively and qualitatively different manner than other types of faces, such as faces of family members, friends, famous people, or unfamiliar people. For example, individuals tend to recognize the own face faster than other faces (e.g., Keenan et al., 2000; Tong & Nakayama, 1999) and this advantage persists even for inverted views (Keyes & Brady, 2010). These findings suggest that the own face is robustly represented in our mind (Tong & Nakayama, 1999). Furthermore, the perception of own face evokes differential electrophysiological and BOLD responses (Alzueta et al., 2019; Devue & Brédart, 2011; Estudillo, 2017; Estudillo et al., 2018). For instance, Keyes et al. (2010) reported a less positive P200 component for the own face compared to both familiar and unfamiliar faces (see also Estudillo, 2017; Estudillo et al., 2018).

Even though holistic face perception supports the processing of both familiar and unfamiliar faces, it is possible that the own face, given its distinct visual experience, might be processed in a qualitatively different manner. For example, although it may be argued that one may also see themselves in photographs and video images, the visual experience of the own face is mostly acquired from self-inspection in mirrors (Brédart, 2003; Gregory, 2001). The distribution of views for one's own face is thus generally mirror-reversed frontal views (Brédart, 2003). The effect of such exposure can be seen through participants’ preference for mirror-reversed images of the own face compared to non-reversed images. This preference is, however, not found in familiar faces (e.g., Brady et al., 2005; Laeng & Rouw, 2001; Troje & Kersten, 1999). Similarly, Brédart (2003) showed that individuals use different types of information to determine the orientation of their own faces and other familiar faces. Specifically, when determining the orientation of their own face, individuals relied preferentially on the location of asymmetric features (e.g., moles, scars, or blemishes). In contrast, observers relied preferentially on facial configural information when determining the orientation of a familiar face. According to Brédart (2003), these differences are explained by the different visual experiences gathered with the self-face and familiar faces.

Furthermore, it is also important to acknowledge that the goal of processing other people's and own faces is different. Specifically, individuals generally perceive the face of other people for identification purposes, whereas the own face is generally viewed for the inspection of facial features through the mirror (e.g., for grooming purposes; see Estudillo & Bindemann, 2017a, 2017b). In other words, although holistic information might be important to make identity or emotion judgements when viewing others’ faces, individuals may naturally pay attention to more subtle facial details when they view themselves in the mirror.

In agreement with this idea, a few studies have questioned the holistic processing of the own face and have proposed a more featural approach. For instance, in a mental imagining study, Greenberg and Goshen-Gottstein (2009) measured the time participants took to generate a mental image of one's own face or a mental image of other people's faces. The authors showed that participants were faster to create a mental image of a facial feature of their own face than a mental image of a facial feature of a familiar face. However, participants were slower to create a mental imagery of the whole own face than a mental image of the whole familiar face. These findings suggest that the processing of the own face relies on featural information.

Nevertheless, this evidence is mainly based on a subjective measure (i.e., participants’ reports of mental imagining of faces). Keyes and Brady (2010) presented evidence from a more objective measure (i.e., the face inversion task). As aforementioned, inverting a face is known to disrupt holistic processing for faces (Rossion, 2008, 2009), resulting in poor recognition of faces. In Keyes and Brady (2010), individuals were faster and more accurate at recognizing their own faces over both friend's and stranger's faces, and, interestingly, these processing advantages were observed for both upright and inverted orientations. This advantage for the own face suggests that the own face is recognized at a more featural level.

Nonetheless, although Keyes and Brady's (2010) results suggest that the own face is processed in a more featural manner, these findings do not deny the holistic nature of face processing. In fact, their study also showed a smaller but significant FIE for the own face. This finding is in agreement with a recent eye-tracking study that showed that the own face is processed in both a featural and holistic manner (Hills, 2018). Given the importance of the own face, this dual-processing strategy for the own face ensures that the own face is efficiently processed (Hills, 2018).

## The Current Study

Although both Greenberg and Goshen-Gottstein (2009) and Keyes and Brady (2010) have presented evidence that the own face may be processed in a more featural manner, the former study presented evidence that is mainly based on a subjective measure (i.e., a mental imagining task), whereas Keyes and Brady (2010) presented evidence from a more objective measure with the face inversion task. However, a limitation of the face inversion task is that the interference of holistic processing is inferred by the FIE, but holistic processing is not directly manipulated in the face inversion paradigm (Tanaka & Simonyi, 2016). Furthermore, it has been suggested that inversion disrupts both holistic and featural processing (see McKone & Yovel, 2009). Finally, the face inversion task is only one of the three measures of holistic face processing and, as previously noted, it is not always associated with other holistic processing tasks, such as the part-whole and composite face task (Rezlescu et al., 2017).

This study thus aimed to extend the findings that the own face may be processed in a more featural manner compared to familiar and unfamiliar faces. This study used the three standard tests of holistic face processing, a face inversion, a composite face, and a part-whole task, to further examine the role of holistic and featural processing when perceiving faces with different levels of familiarity. We hypothesized that the own face is processed in a more featural manner, whereas both familiar and unfamiliar faces are processed in a more holistic manner.

To test for these hypotheses, participants were asked to identify their face, a friend's face, and an unfamiliar face in three different tasks. In the face inversion task, participants were asked to identify the three faces presented in either upright or inverted conditions. In the composite face task, participants were required to identify the top halves of the faces presented either in aligned or misaligned conditions with a distractor bottom part. Lastly, in the part-whole task, participants viewed facial features presented in either isolation or a whole foil face context, and they were asked to decide the identity to whom the facial features belong.

If the own face is processed in a more featural manner compared to the familiar and the unfamiliar faces, we would expect a smaller face inversion, composite face, and part-whole effects for the own face compared to those of the familiar and unfamiliar faces. More specifically, for the face inversion task, the differences between the identification of upright and inverted faces should be smaller for the own face compared to these differences for familiar and unfamiliar faces. For the composite face task, the self-face would be less affected by the “holistic interference” from the to-be-ignored bottom half (see Rossion, 2013) compared to the familiar and unfamiliar faces. Lastly, for the part-whole task, we would expect weaker “whole-face interference” during part recognition for the own face features (see Tanaka & Simonyi, 2016). That is, compared to familiar and unfamiliar faces, the recognition of a specific face part of the own face embedded in the context of a foil face will be less affected by the presence of the foil face's facial features.

## Method

### Participants

Fifty Malaysian Chinese (13 males) participants whose ages ranged between 18 and 20 years old (*M* = 20.9, *SD* = 1.78) were recruited from the University of Nottingham Malaysia. A power analysis performed in G*Power 3.1 (Faul et al., 2007) with an effect size of 0.15 and an alpha of .05 gives a required sample size of 50 participants to achieve 80% power. This experiment utilized a 3 (*target identity: self, friend, or unfamiliar*) × 2 (*orientation: inverted or upright; alignment: aligned or misaligned; or whole part: whole or part*) within-subjects design.

Participants were recruited in pairs of friends matched by age, gender, and race so that each of them served as a friend of the other participant. The age range allowed for matching is up to three years. Participants were either awarded with course credits or compensated financially for their participation. Ethics approval for this study was obtained from the Science and Engineering Research Ethics Committee of the University of Nottingham Malaysia (JLKW220520).

### Stimuli

#### Image collection

**Figure A1. fig5-20416695221111409:**
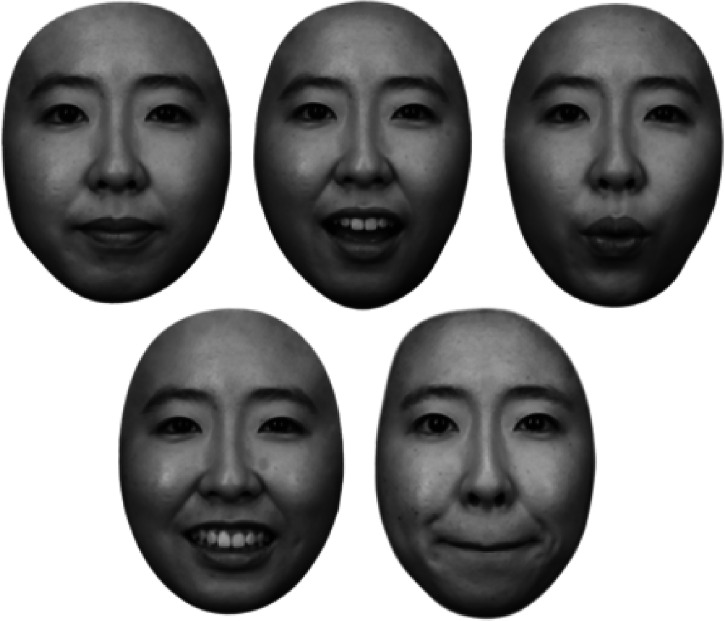
Example of face images. *Note*. Example of five different images for each identity. From the top left: “neutral,” “A,” “O,” “E,” and “M” expressions. All images were cropped based on their individual contours and converted to grayscale.

Across all three tasks, photograph stimuli (self-face and friend face) were individually tailored to each participant. Each participant was photographed under similar conditions (i.e., constant lighting and uniform background), in a frontal position while assuming a neutral expression and while articulating four different speech sounds (e.g., A, O, E, and M; see Figure A1). Different images were used for each identity to reduce image-specific effects (Bruce, 1982; Estudillo, 2012; Estudillo & Bindemann, 2014). All different images were used as “self-face” for the participant themselves and as “friend's face” for their friend, respectively.

Six separate individuals (three males and three females) matched in age were photographed under the same conditions to be used as unfamiliar targets. Different unfamiliar faces were used for each task (counterbalanced across each participant) to reduce the learning of the unfamiliar face. Specifically, in the learning stage, participants were asked to learn only one out of the three unfamiliar faces across each task and the learned faces were presented in a neutral expression. The face images with different expressions (e.g., neutral, A, O, E, and M) were used across all three tasks. All images were collected and processed at least one week prior to the experimental session.

#### Image Processing

To prepare the images for this experiment, all face stimuli were edited using Adobe Photoshop CS6. All original photographs were resized to 397 × 567 pixels (16-bit depth), corresponding to an approximate visual angle of 9.53˚ horizontally and 13.58˚ vertically at a viewing distance of 63 cm. Moreover, in an effort to eliminate any low-level variations between different types of stimuli, Gaussian (radius = 3 pixels) and Pixelate Filters (cell size = 2 square) in Photoshop^TM^ were applied to all face images to normalize the image resolution. Across all the three tasks, all photographs were aligned on the eyes’ position and were cropped based on their individual contours and external features (i.e., hairs and ears were removed). Finally, all face images were also converted to greyscale. In addition to minimizing the differences in non-facial cues, these transformations are important in providing the most optimal stimuli for holistic face processing (see Retter & Rossion, 2015). “Self-face” images were presented in a mirror-reversed orientation (i.e., the view in which people generally view their own face), whereas the “friend” and “unfamiliar” images were presented in a normal orientation.

### Procedure

Participants were asked to complete three experimental tasks in a counterbalanced order. Participants were seated approximately 63 cm from the screen. The screen measured horizontally 51 cm and vertically 28.5 cm. Participants were instructed to respond as quickly and accurately as possible.

#### Face Inversion Task

**Figure 1.**
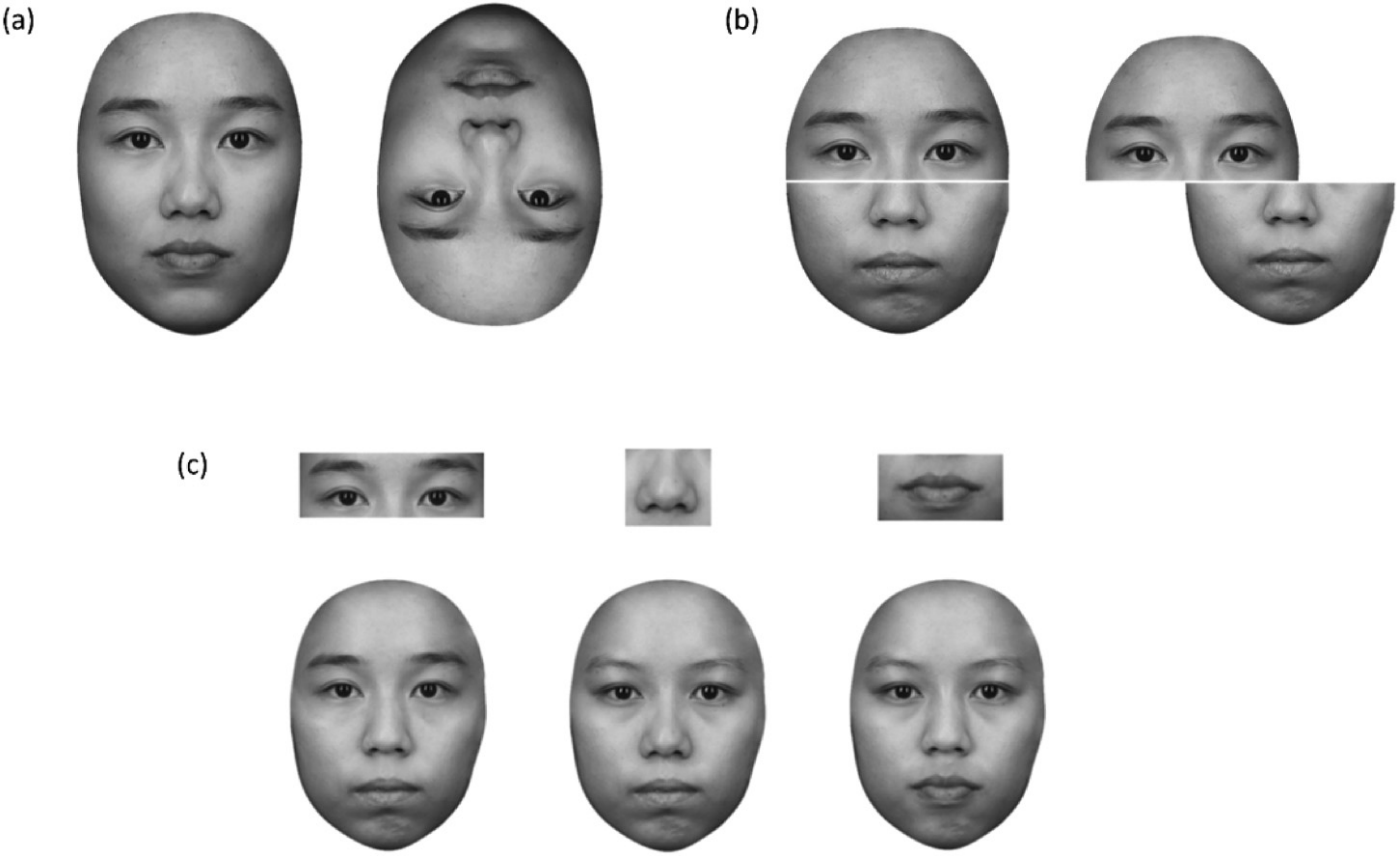
Examples of stimuli used in each task. *Note.* (a) In the inversion task, face images are presented in an upright manner (left) and an inverted manner (right) by flipping them vertically downwards; (b) in the composite face task, the top half (e.g., of self-face) and the bottom half (e.g., of a novel face) combined to form a face composite in an aligned (left) and misaligned (right) manner with a three-pixel gap; (c) in the part-whole task, facial features in “part” condition (top) and face foils created by substituting one critical feature across a face template, while keeping the original configuration of the face template (bottom).

Each face image had an upright and inverted version (see Figure 1a). Inverted face images were created by flipping the upright face images vertically downwards.

This task had two within-subject variables: target identity (self, friend, and unfamiliar) and face orientation (upright and inverted). Each participant was asked to complete 12 practice trials and two blocks of 120 experimental trials. The 240 trials included 80 trials for each identity (self, friend, and unfamiliar), with 40 upright trials (5 different images × 8 repetitions) and 40 inverted trials (5 different images × 8 repetitions). All 240 experimental trials were randomized.

**Figure A2. fig6-20416695221111409:**
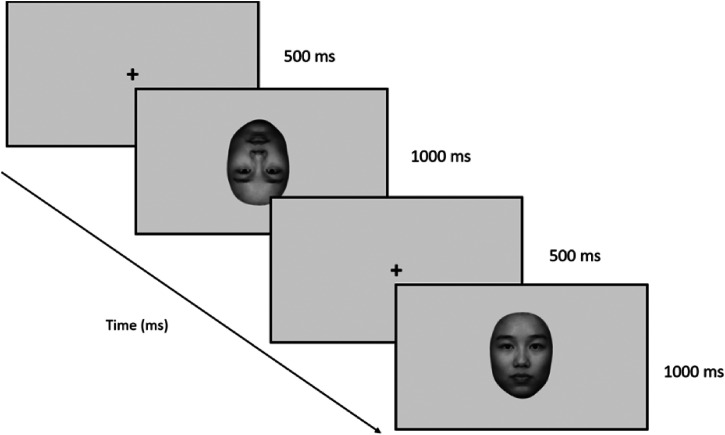
Experimental paradigm of the face inversion task. *Note*. Experimental paradigm: face inversion task started with the learning stage for an unfamiliar face. On each trial, a central fixation was replaced by either an upright or inverted face stimulus (self-face, friend's face, or unfamiliar face) after 500 ms.

This task consisted of an initial learning stage for the unfamiliar face and a subsequent identification stage with all three faces. Observers were asked to memorize an unfamiliar face with the given name (e.g., “Wong”). This learning stage was self-paced. Observers pressed a key to move on to the identification stage once they indicated that they were ready (for a similar procedure, see Estudillo et al., 2018; Tanaka et al., 2006). The learned unfamiliar face was the same as the one shown in the identification task. In the identification task, participants were asked to indicate the identity of the presented face, with the following instructions: “If it is your own face, press the ‘J’ key; if it is your friend's face, press the ‘K’ key; and if it is Wong's face, press the ‘L’ key”. Each trial was then initiated with a central fixation cross appearing for 500 ms. The fixation cross was replaced by an image of an upright or inverted self-face, friend's face, or unfamiliar face, which remained on screen for 1000 ms or until participants made a response. The response time and whether the response was correct were recorded. See Figure A2 for a depiction of the experimental paradigm.

#### Composite Face Task

In this task, participants were asked to identify the top half of the composite face presented in either an aligned or misaligned condition with an unfamiliar face. Face composites were first created by halving face images horizontally across the middle of the nose. Next, the top half of a face (from self, friend, or unfamiliar face) was combined with the bottom half of another face from a set of novel faces (either 12 females or 12 males), either in an aligned manner or misaligned manner, with a gap of 0.5% of the image height separating the top and bottom face halves (see Figure 1b). This gap aimed to provide an objective definition of the to-be-matched face half and to remove an enhanced border contrast (see Rossion & Retter, 2015). Specifically, aligned conditions were when the top and bottom halves of a face were combined to form a standard face template, whereas, in misaligned conditions, the bottom half of a face was moved to the left/right by half of the face width. The bottom halves of the face composites were always different. Specifically, each identity (self, friend, or unfamiliar) was combined with bottom halves from four different novel faces, with five different images of a novel face (i.e., the different expressions: A, O, E, M, and neutral). The bottom and top halves were also matched in terms of the speech sound (e.g., the self-face with “A” expression was matched with a novel-face with “A” expression). The novel faces used for each identity were randomized across participants.

**Figure A3. fig7-20416695221111409:**
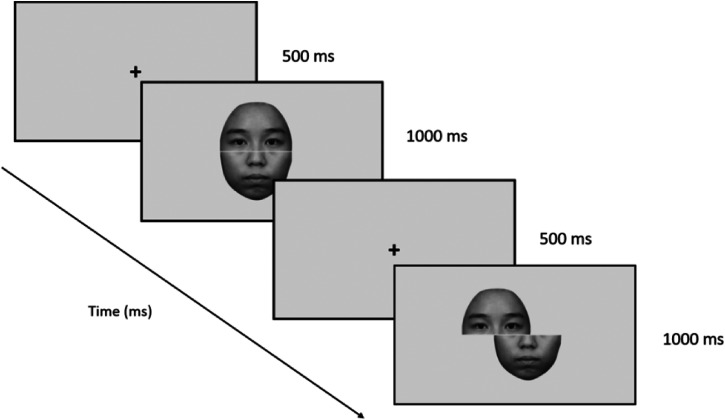
Experimental paradigm of the composite face task. *Note*. Experimental paradigm: composite face task started with the learning stage for an unfamiliar face. On each trial, a central fixation was replaced by an aligned or misaligned face stimulus (self-face, friend's face, or unfamiliar face) after 500 ms.

This task had two within-subject variables: target identity (self, friend, and unfamiliar) and face alignment (aligned and misaligned). Each participant was asked to complete 12 practice trials and two blocks of 120 experimental trials. For each block, there was a total of 60 aligned trials (3 identities × 20 repetitions) and 60 misaligned trials (3 identities × 20 repetitions). All 240 trials were randomized. The procedure was identical to the procedure of the face inversion task: with a learning stage for unfamiliar faces followed by the identification stage for all faces, but in this task, participants were asked to identify the top halves of face stimuli and to ignore the bottom halves. See Figure A3 for a depiction of the experimental paradigm.

#### Part-Whole Task

In this task, participants were asked to identify facial features presented in either isolation or the context of a whole foil face, and they were asked to decide which identity the facial feature belongs to (e.g., “whose eyes are these?” or “whose nose is this?”). For the whole face condition, face foil stimuli were created by swapping only one critical facial feature (eyes, nose, or mouth) of a target face (self, friend, or learned unfamiliar face) across six novel faces (3 females) of the same gender, while keeping the original configuration of the novel face template. In other words, the target face and whole foil faces were similar with the exception of the critical facial feature under examination (see Figure 1c). For instance, for the recognition of eyes in the whole face condition, the non-target features (nose and mouth of the novel face) were kept unchanged. Altogether, there was a total of nine whole foil faces and nine face parts stimuli for each participant: for each identity, three whole face foils were formed by swapping one of the critical facial features (eyes, nose, or mouth) with a novel face whereas three face parts were created with only one critical facial feature (eyes, nose, or mouth).

**Figure A4. fig8-20416695221111409:**
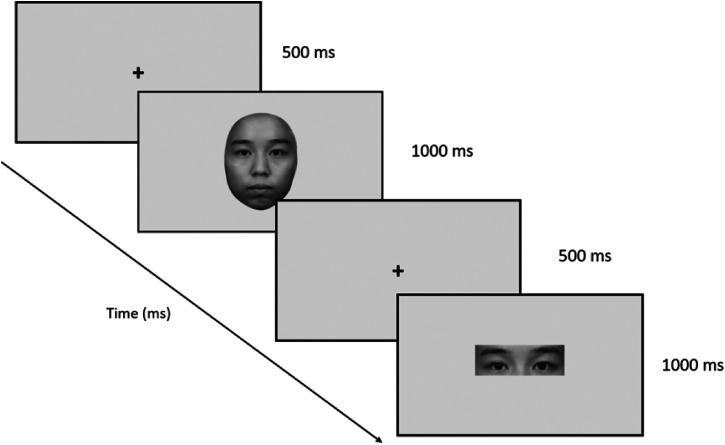
Experimental paradigm of the part-whole task. *Note.* Experimental paradigm: part-whole task started with the learning stage for an unfamiliar face (top); on each trial, a central fixation was replaced by either “part” or “whole” stimuli after 500 ms.

This task had two within-subject variables: target identity (self, friend, and unfamiliar) and part-whole condition (whole or part). Each participant was asked to complete 12 practice trials and a total of 180 trials. There were three feature blocks (eyes, nose, or mouth), each with 30 upright “whole” trials and 30 upright “part” trials respectively. Similar to the face inversion and composite face tasks, observers first went through a familiarization stage for the unfamiliar face followed by the identification stage, except that participant was asked to indicate the identity of the facial features presented either in isolation or in a whole foil face context. See Figure A4 for a depiction of the experimental paradigm.

### Data Analyses

As holistic processing is measured by the face inversion, composite face, and PWEs, each of these effects was computed by comparing the accuracy score and median reaction time (RT) from the baseline conditions (i.e., upright, aligned, or whole) and the conditions of interest (i.e., inverted, misaligned, or part). The median RT was used instead of the mean RT to remove the influence of extreme values.

If the own face is processed more in a more featural manner compared to a familiar and an unfamiliar face, we would then expect a smaller face inversion, composite face, and PWEs for the own face than for those of the familiar and unfamiliar faces. Specifically, for the face inversion task, we expected the differences between the identification of upright and inverted faces to be smaller for the own face compared to these differences for familiar and unfamiliar faces. In this case, the FIE was calculated by considering both the accuracy and median RT for correct responses on the upright and inverted trials, with the formula: (accuracy: upright–inverted; median RT: inverted–upright).

In addition, for the composite face task, we expected the self-face to be less affected by the “holistic interference” from the to-be-ignored bottom half compared to the familiar and unfamiliar faces. In this instance, the CFE was computed by considering the accuracy scores and median RT for correct responses on the aligned and misaligned trials, with the formula: (accuracy: misaligned–aligned; median RT: aligned–misaligned).

Lastly, for the part-whole task, we expected the recognition of a face part of the own face to be less affected by the presence of other facial features in a whole foil face context (i.e., less whole-face interference) compared to the recognition of a face part of familiar and unfamiliar faces. In this case, the PWE was calculated by considering the accuracy scores and median RT of correct responses in the “whole” and “part” conditions, with the formula: (accuracy: part–whole; median RT: whole–part) (see Rezlescu et al., 2017; Rossion, 2013).

Next, for each task, 3 (*target identity: self, friend, or unfamiliar*) × 2 (*orientation: inverted or upright; alignment: aligned or misaligned; part-whole: whole or part*) repeated-measures analyses of variance (ANOVAs) and follow-up comparisons were performed on the accuracy scores and median RTs respectively. In addition, to further explore the interaction effects of identity and FIE, CFE, and PWE, a one-way ANOVA was conducted on the accuracies and median RT for each identity. Overall, we expected that (a) for the face inversion task, participants would show a weaker FIE for the own face as compared to the friend and unfamiliar faces; (b) for the composite face task, participants would show a weaker CFE for the own face than for the friend and unfamiliar faces; and (c) for the part-whole task, participants would show a weaker PWE for the own face as compared to the friend and unfamiliar faces.

## Results

### Face Inversion Task

#### Accuracy

**Figure 2.**
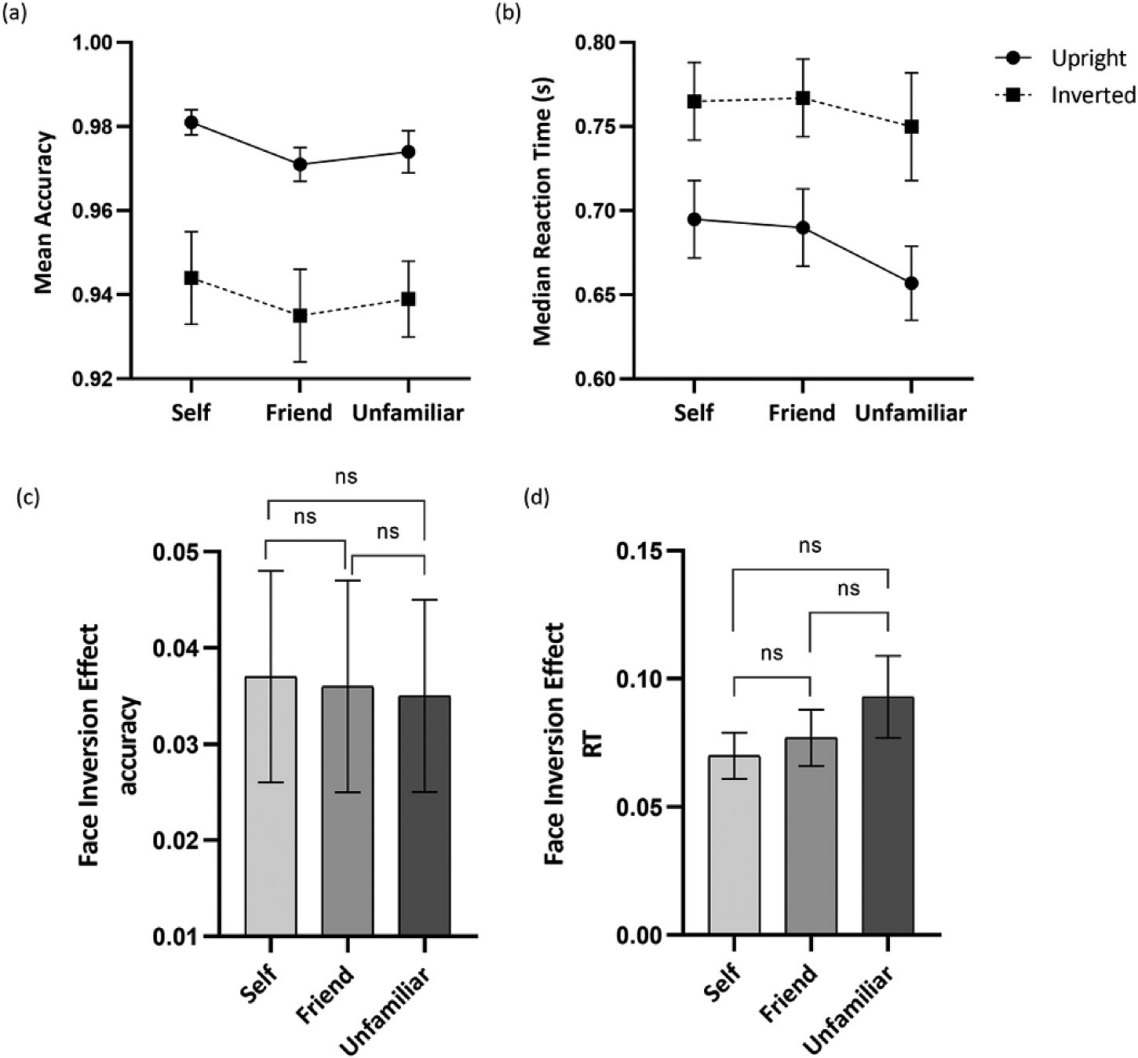
(a) Mean accuracies and (b) median RT in response to identifying self, friend, and unfamiliar faces in upright (circles) and inverted (squares) conditions; (c) the FIE on mean accuracies and (d) median RT for self, friend, and unfamiliar faces. *Note*. Error bars represent the standard error of the mean. FIE = face inversion effect; RT = reaction time.

Figure 2a shows the accuracy for each face identity across upright and inverted conditions. The analysis revealed a significant main effect of orientation, *F*(1, 49) = 30.14, *p* < .001, *η_p_*^2^ *=* 0.381, with a higher accuracy reported for face images presented in upright condition (*M* = 0.975, *SD* = 0.003) compared to inverted condition (*M* = 0.939, *SD* = 0.007). The analysis revealed no other significant main or interaction effects.

#### Median RT

Figure 2b shows the median RT for each face identity across upright and inverted conditions. The analysis revealed a significant main effect of identity, *F*(2, 98) = 4.84, *p* = .010, *η_p_*^2^ *=* 0.090. Holm–Bonferroni post hoc comparisons indicated that participants performed significantly faster for an unfamiliar face compared to the friend's face (*p* = .008, *d* = −0.45), whereas there were no significant differences in the RT between the self-face and friend face (*p* = 1, *d* = −0.02) and the self-face and unfamiliar face (*p* = .051, *d* = −0.35). Next, a significant main effect of orientation was reported as well, *F*(1, 49) = 85.61, *p* < .001, *η_p_*^2^ *=* 0.636, with participants performing faster on the upright trials (*M* = 0.680, *SD* = 0.022) compared to the inverted trials (*M* = 0.761, *SD* = 0.027). The analysis further revealed no significant interaction effect.

#### FIE

Figure 2c and 2d shows the FIE for both accuracy and median RT across each face identity. For accuracy, the analysis revealed no significant main effect of identity, *F*(2, 98) = 0.01, *p* = .990, *η_p_*^2^ *=* 0.000. Similarly, for median RT, the analysis indicated no significant main effect of Identity, *F*(2, 98) = 1.28, *p* = .282, *η_p_*^2^ *=* 0.025.

### Composite Face Task

#### Accuracy

**Figure 3.**
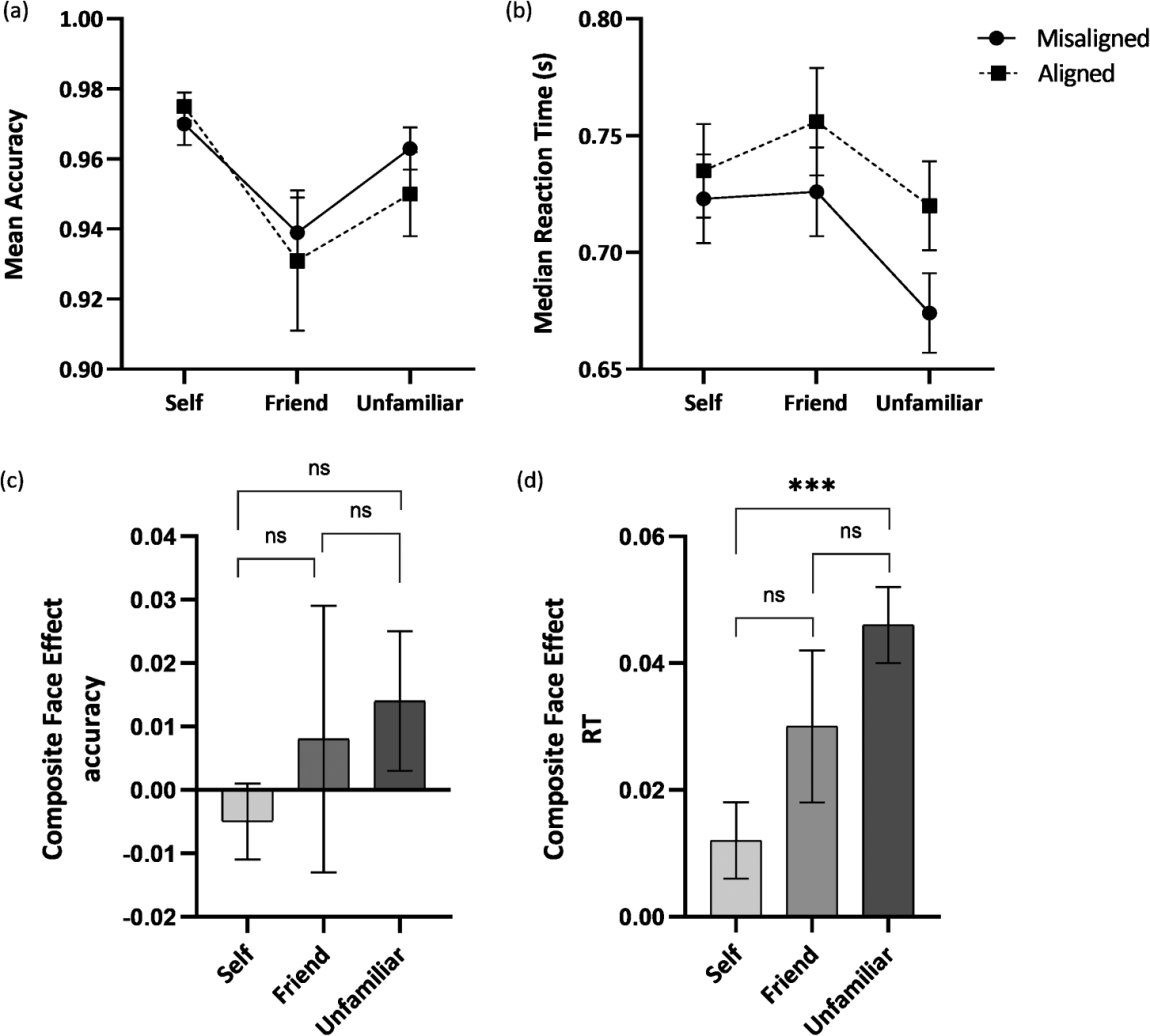
(a) Mean accuracies (b) and median RT in response to identifying self, friend, and unfamiliar faces in misaligned (circles) and aligned (squares) conditions; (c) the Composite Face Effect (CFE) on mean accuracies and (d) median RT for self, friend, and unfamiliar faces. *Note*. Error bars represent the standard error of the mean. RT = reaction time.

Figure 3a shows the accuracy for each identity across misaligned and aligned conditions. The analysis revealed a significant main effect of identity, *F*(2, 98) = 8.77, *p* < .001, *η_p_*^2^ *=* 0.152. Holm–Bonferroni post hoc comparisons indicated that participants performed better for the self-face compared to a friend's face (*p* = .006, *d* = 0.46) and for an unfamiliar face compared to a friend's face (*p* = .016, *d* = −0.41), but there were no significant differences in the accuracy for self-face and unfamiliar face (*p* = .115, *d* = 0.30). The analysis further revealed no other significant main or interaction effects.

#### Median RT

Figure 3b shows the median RT for each face identity across misaligned and aligned conditions. The analysis revealed a significant main effect of identity, *F*(2, 98) = 12.32, *p* < .001, *η_p_*^2^ *=* 0.201. Holm–Bonferroni post hoc comparisons revealed that participants responded faster for unfamiliar faces compared to self-face (*p* = .012, *d* = 0.43) and friend's face (*p* < .001, *d* = 0.74), whereas there were no significant differences in the RT for self-face and friend's face (*p* = .467, *d* = −0.20). In addition, a significant main effect of Alignment was reported, *F*(1, 49) = 31.98, *p* < .001, *η_p_*^2^ *=* 0.395, with participants performing faster on misaligned trials (*M* = 0.708, *SD* = 0.018) compared to aligned trials (*M* = 0.737, *SD* = 0.019). Lastly, the analysis revealed a significant interaction effect between identity and alignment, *F*(1.48, 72.64) = 3.91, *p* = .036, *η_p_*^2^ *=* 0.074 (Huynh–Feldt corrected). Holm–Bonferroni post hoc comparisons revealed that for the self-face, there were no significant differences in the RT for misaligned and aligned trials (*p* = .066, *d* = −0.27), whereas participants performed faster for misaligned trials compared to aligned trials for both friend's face (*p* = .019, *d* = −0.34) and unfamiliar face (*p* < .001, *d* = −1.01).

#### CFE

Figure 3c and 3d shows the CFE for both accuracy and median RT across each face identity. For accuracy, the analysis revealed no significant main effect of Identity, *F*(2, 98) = 0.59, *p* = .554, *η_p_*^2^ *=* 0.012. On the other hand, for the median RT, the analysis revealed a significant main effect of identity, *F*(2, 98) = 4.42, *p* = .023, *η_p_*^2^ *=* 0.074. Holm–Bonferroni post hoc comparisons revealed that the CFE was significantly smaller for the self-face compared to an unfamiliar face (*p* < .001, *d* = −0.58), whereas there were no significant differences in the CFE for self-face and friend's face (*p* = .726, *d* = −0.17) and for friend and unfamiliar face (*p* = .573, *d* = −0.19).

### Part-Whole Task

#### Accuracy

**Figure 4.**
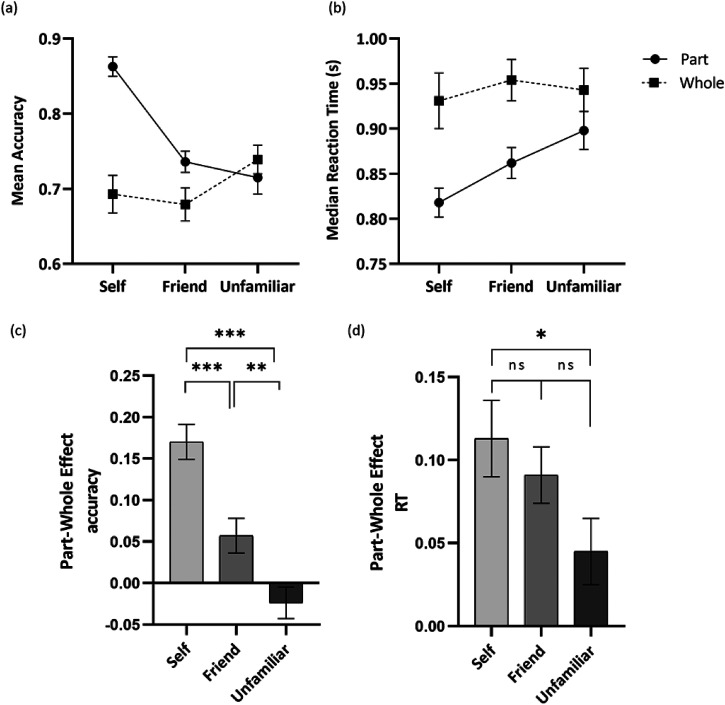
(a) Mean accuracies (b) and median RT in response to identifying self, friend, and unfamiliar faces in part (circles) and whole (squares) conditions; (c) the PWE on mean accuracies and (d) median RT for self, friend, and unfamiliar faces. *Note*. Error bars represent the standard error of the mean. PWE = part-whole effect; RT = reaction time.

Figure 4a shows the accuracy for each face identity across part and whole conditions. The analysis revealed a significant main effect of identity, *F*(2, 98) = 8.29, *p* < .001, *η_p_*^2^ *=* 0.145. Holm–Bonferroni post hoc comparisons revealed that participants performed better for self-face compared to friend's face (*p* < .001, *d* = 0.64) and unfamiliar face (*p* = .052, *d* = 0.34), whereas there were no significant differences in the accuracy for friend's face and unfamiliar face (*p* = .735, *d* = −0.17). A significant main effect of part-whole was also reported, *F(*1, 49) = 28.77, *p* < .001, *η_p_*^2^ *=* 0.370, with a higher accuracy for part trials (*M* = 0.771, *SD* = 0.012) compared to whole trials (*M* = 0.704, *SD* = 0.017). The analysis further revealed a significant interaction effect for identity and part-whole, *F*(2, 98) = 24.75, *p* < .001, *η_p_*^2^ *=* 0.336. Holm–Bonferroni post hoc comparisons revealed that for both self-face (*p* < .001, *d* = 1.17) and friend's face (*p* = .009, *d* = 0.38), participants performed better on part trials compared to whole trials whereas for unfamiliar face, there was no significant difference between part and whole trials (*p* = .229, *d* = −0.17).

#### Median RT

Figure 4b shows the median RT for each face identity across part and whole conditions. The analysis revealed a significant main effect of identity, *F*(2, 98) = 7.05, *p* < .001, *η_p_*^2^ *=* 0.126. Holm–Bonferroni post hoc comparisons indicated that participants performed faster for self-face compared to friend's face (*p* = .045, *d* = −0.36) and unfamiliar face (*p* = .003, *d* = −0.50), whereas there were no significant differences in the RT for friend's face and unfamiliar face (*p* = .843, *d* = −0.15). In addition, a significant main effect of part-whole was reported, *F*(1, 49) = 33.04, *p* < .001, *η_p_*^2^ *=* 0.403, with participants performing faster in the parts condition (*M* = 0.860, *SD* = 0.016) compared to the whole condition (*M* = 0.943, *SD* = 0.024). Finally, the analysis revealed a significant interaction effect for identity and part-whole, *F*(2, 98) = 4.02, *p* = .021, *η_p_*^2^ *=* 0.076. Holm–Bonferroni post hoc comparisons revealed that for all faces, participants performed faster in the part condition compared to the whole condition (all *p*s < .001).

#### PWE

Figure 4c and 4d shows the PWE for both accuracy and median RT across each face identity. For accuracy, the analysis revealed a significant main effect of Identity, *F*(2, 98) = 24.75, *p* < .001, *η_p_*^2^ *=* 0.336. Holm–Bonferroni post hoc comparisons revealed a larger PWE for the self-face compared to a friend's face (*p* < .001, *d* = 0.63) and an unfamiliar face (*p* < .001, *d* = 0.86), whereas the friend's face also showed a larger PWE compared to an unfamiliar face (*p* = .007, *d* = 0.45). Finally, for the median RT, the analysis revealed a significant main effect of identity, *F*(2, 98) = 4.02, *p* = .021, *η_p_*^2^ *=* 0.076. Holm–Bonferroni post hoc comparisons revealed a trend of a larger PWE for self-faces compared to unfamiliar faces (*p* = .051, *d* = 0.35), whereas there were no significant differences in the PWE for self and friend's face (*p* = 1, *d* = 0.14) and for friend and unfamiliar face (*p* = .136, *d* = 0.29).

## Discussion

With three standard but relatively independent measures of holistic face processing, we explored the role of holistic and featural processing when perceiving the own, a personally familiar, and an unfamiliar face. Due to the distinct visual experience with the own face, we hypothesized that the own face is processed in a more featural manner whereas both familiar and unfamiliar faces are processed in a more holistic manner. More specifically, if the own face is processed more in a more featural manner, we would expect a smaller inversion, composite, and PWEs for the own face compared to those of the familiar and unfamiliar faces.

Findings in this study could be summarized in the following points: (a) all faces, regardless of their identity, were equally affected by inversion, such that there were no significant differences in the inversion effect between the self-face, friend's face, and unfamiliar face; (b) compared to a friend and an unfamiliar face, the self-face was not affected by the alignment effect and a significantly smaller CFE was observed for the self-face compared to the unfamiliar face; and finally (c) a feature advantage for the self-face compared to other faces was reported, wherein self-face features were better identified when presented in isolation compared to when presented in a whole-face context, but there were no differences in the recognition performance between the two conditions for other faces.

### Self-face Recognition in a Face Inversion Task

With the inversion task, our findings indicated an evident inversion effect for all faces, regardless of their identity, wherein participants overall were poorer and slower in identifying inverted faces compared to upright faces. Specifically, there were no significant differences in the inversion effect across the self, friend, and unfamiliar faces. This finding is consistent with the mounting existing evidence showing that inversion impairs face identification (e.g., Rossion, 2008; Rossion & Gauthier, 2002). As inverting a face disrupts the ability to perceive the face as an integrated whole (Van Belle et al., 2010a; but see McKone & Yovel, 2009), observers would extract facial information from an inverted face in a more featural manner to compensate for this disruption (Barton et al., 2006), and hence amounting to an overall longer response time and lower accuracy when identifying inverted faces compared to upright faces.

More importantly, contradicting our hypothesis, our findings showed that the self-face is not more resistant to the inversion effects compared to other faces. Notably, even after controlling for familiarity effects, we did not observe a smaller inversion effect for one's own face compared to a friend and unfamiliar face. Although our findings contradicted studies which showed that self-face is less vulnerable to inversion effects (e.g., Keyes, 2012; Keyes & Brady, 2010), our findings mirrored findings from a recent study (Alzueta et al., 2021), which presented behavioral and neural evidence that the self-face and friend's face are equally affected by inversion effects. With such findings, Alzueta et al. (2021) argued that the processing advantage for the own face could be better accounted for by the prioritization of one's attentional system rather than a different perceptual mechanism. It is also worth noting that although we pay more attention to the facial features of the own face, we, however, still have the most experience with upright faces; an inverted face does not fit the face template as the first-order configuration is disrupted (e.g., Rossion & Boremanse, 2008), hence reducing the efficiency of extracting facial information from an inverted face (i.e., less effective eye fixation patterns; Hills et al., 2013; Xu & Tanaka, 2013).

On the other hand, it is also worth noting that the inversion task reflects a type of holistic processing that is qualitatively different to that observed in the part-whole and composite tasks. This is supported by recent research that showed that these three measures are, at best, only poorly associated with each other (Rezlescu et al., 2017). Alternatively, it is also possible that inversion might reduce overall processing efficiency and disrupt the ability to extract relevant identity information without affecting whether the faces are being processed in a holistic manner (e.g., Richler et al., 2011; Sekuler et al., 2004; Willenbockel et al., 2010).

Finally, extending on the findings from Alzueta et al.’s (2021) study, we show that familiar faces (self and friend) are not more vulnerable to the inversion effect compared to unfamiliar faces. In other words, our findings seem to suggest that inversion affects the familiar and unfamiliar faces in a similar manner. This finding is rather surprising as studies have demonstrated that due to the importance and increased reliance on holistic processing for familiar faces (see Buttle & Raymond, 2003; Veres-Injac & Persike, 2009), inverting a face stimulus should be more disruptive for familiar faces compared to unfamiliar faces (e.g., Caharel et al., 2006; Keyes & Brady, 2010; but see Tong & Nakayama, 1999). Overall, in the context that the interference of holistic processing is inferred by the inversion effect, our findings seem to suggest that the self-face is not processed in a more featural manner (or lesser use of holistic processing) compared to other faces.

### Self-face Recognition in a Composite Face Task

In the composite face task, participants were asked to identify to whom the top part of the face stimulus belonged with the face stimuli presented in either misaligned or aligned conditions. When identifying the top part of the face stimuli as one's own face, participants performed similarly across the aligned and misaligned conditions in terms of RT. However, participants were quicker to identify the friend and unfamiliar face when the top part is presented in a misaligned condition compared to in an aligned condition. In other words, compared to the friend and unfamiliar face, the self-face is less affected by the “holistic interference” (Rossion, 2013) from the to-be-ignored bottom half.

In a composite face task (Young et al., 1985), the composite effect indexes a failure of selectively attending to just one half of the face as the faces are processed as undifferentiated wholes and observers cannot ignore the task-irrelevant face half (Richler & Gauthier, 2014; Richler et al., 2008). In other words, there is a “holistic interference” from the task-irrelevant face half as faces are processed as wholes (Rossion, 2013). Therefore, based on the expectation that the composite effect measures holistic processing, it is appealing to infer from our findings that one's own face is processed in a more featural manner compared to other faces as the self-face is seemingly less affected by the “holistic interference” from the task-irrelevant face half.

Nevertheless, our findings need to be treated with caution as further analyses showed that although there was a significantly smaller CFE for the self-face compared to an unfamiliar face, there were no significant differences in the CFE between the self-face and a friend's face. One could argue that mere familiarity effects can explain this pattern of findings, but we wish to point out that no significant differences were reported for the CFE between a friend and an unfamiliar face.

### Self-face Recognition in a Part-Whole Task

In the part-whole task, participants were shown facial features (eyes, nose, and mouth) presented in isolation or in a whole-face context and were asked to identify to whom the facial feature belonged. Overall, participants showed better and faster recognition of one's own face facial features presented in isolation compared to when presented in a whole-face context. However, there were no differences in the behavioral performance for the identification of friends and unfamiliar facial features presented in isolation or in a whole-face context. Thus, our results show a feature advantage for the self-face compared to friends and unfamiliar faces.

With the part-whole task, we wanted to explore whether the recognition of a specific face part of the own face (i.e., eyes) would be less affected by the presence of other facial features (i.e., nose and mouth) in a foil whole-face context. In other words, we expected to find smaller differences between part and whole trials for the own face compared to both the familiar and the unfamiliar faces. Interestingly, and in contrast to our hypothesis, the own face produced stronger PWEs compared to the other faces. Therefore, irrelevant facial features from a foil face seem to have stronger interference effects on the own facial features. However, the fact that this effect occurs as a consequence of a larger feature advantage (i.e., better performance for isolated features) for the own face compared to both friend and the unfamiliar face (see Figure 4) rules out that this PWE reflects stronger holistic processing for the own face. Instead, the stronger interference from irrelevant facial features in the identification of the own facial features might reflect congruency effects (Leder & Carbon, 2005). This congruency effect would arise because people have a stronger representation of their individual facial features (Greenberg & Goshen-Gottstein, 2009). Thus, when one's facial features are presented in the context of a foil face, there is a contextual mismatch with the way that these features are normally processed. On the contrary, as the representation of other people's facial features is much weaker (Greenberg & Goshen-Gottstein, 2009), this congruency effect would be smaller. Although this explanation is only tentative, it is supported by previous research showing that a facial feature learned in isolation is subsequently better recognized when presented in isolation than in the context of a whole face (Leder & Carbon, 2005).

### Self-face Recognition, Holistic Processing, and Featural Processing

Overall, findings from this study showed that compared to a friend or an unfamiliar face, the self-face seemed to be processed in a more featural manner. This is reflected by a smaller “holistic interference” by a task-irrelevant bottom half face for the own face compared to other faces in the composite face task and a stronger feature advantage for the own face compared to other faces in the part-whole task. Nevertheless, we observed that like other faces, the self-face is also affected by inversion.

Firstly, these findings suggest that the own face uses both holistic and featural processing. Given the significance of the own face, this dual-processing strategy for the own face ensures that the self-face is processed efficiently (see Hills, 2018). For example, although Keyes and Brady (2010) suggested that the own face is processed in a more featural manner, findings from their study, however, did not disregard the holistic processing nature of face processing as they reported a smaller but significant FIE for the own face. Similarly, in our study, we also showed that the recognition of the self-face is affected by inversion in a similar manner as other faces, suggesting that self-face recognition also relies on holistic processing.

Nonetheless, we wish to point out that although the inversion task has been suggested to disrupt holistic processing in a more naturalistic way (Alzueta et al., 2021) and is deemed to be the best in predicting face recognition abilities (Rezlescu et al., 2017), these findings do not imply that holistic processing is directly manipulated in the face inversion task (Tanaka & Simonyi, 2016). In fact, some authors have argued that inversion disrupts both holistic and featural processing (see McKone & Yovel, 2009) and simply reduces the overall processing efficiency to extract relevant facial information rather than having a qualitative change in the processing of an inverted face (Sekuler et al., 2004; Willenbockel et al., 2010). Hence, findings from the face inversion task need to be interpreted with caution.

Our findings also showed that different tasks reflect different results in terms of holistic processing of self-faces. Specifically, there was no interaction between inversion and face types but there was an interaction between alignment and face types and between part-whole and face types. When interpreting findings from this study, one should take into consideration that the inversion, composite, and PWEs are poorly associated with each other, and these three measures might be tapping into different perceptual mechanisms (see Rezlescu et al., 2017; Richler et al., 2012). For instance, with the composite task, holistic processing is indexed by a holistic interference: individuals fail to selectively attend to just the task-relevant top half of the face (Richler & Gauthier, 2014; Rossion, 2013); and with the part-whole task, holistic processing is generally demonstrated by showing that facial features are encoded and integrated as one “whole” perceptual unit (i.e., an upright face) rather than being encoded as isolated facial features (Tanaka & Farah, 1993). Hence, considering that the inversion, the composite, and the part-whole tasks might be measuring different forms of holistic processing and the measure of holistic processing may be affected by the different task manipulations, future studies could consider the use of a gaze-contingent window (Van Belle et al., 2010b) to explore the role of holistic and featural processing for the own face as this technique provides a clear and direct demonstration whether the observer's perceptual field comprises one facial feature at a time or the whole face.

## Conclusions

To our knowledge, this is the first attempt to explore the role of holistic and featural processing in the identification of the own face using three standard, but largely independent measures of holistic face processing: the FIE, the CFE, and part-whole interference or effect. Our findings show that (a) the own face is not less resistant to inversion than other faces; (b) a smaller composite effect (or less holistic interference) was reported for the own face compared to an unfamiliar face; and lastly (c) a feature advantage (i.e., better recognition for isolated features compared to in a whole-face context) was found for the own face compared to other faces. In sum, findings from this study seem to suggest that not all faces are processed similarly. Specifically, the own face seems to be processed in a more featural manner but also relies on holistic processing compared to other familiar and unfamiliar faces. Our findings also suggest that caution is needed when designing and interpreting face perception studies involving the own face and other faces as these faces seem to be processed differently. Finally, this work also highlights the importance of taking into consideration how different experimental manipulations could affect the measure of holistic processing.

## Appendix

[Fig fig5-20416695221111409] [Fig fig6-20416695221111409] [Fig fig7-20416695221111409] [Fig fig8-20416695221111409]

## References

[bibr1-20416695221111409] Alzueta E. Kessel D. Capilla A. (2021). The upside-down self: One's own face recognition is affected by inversion. Psychophysiology, 58(12), e13919. 10.1111/psyp.13919 34383323

[bibr2-20416695221111409] Alzueta E. Melcón M. Poch C. Capilla A. (2019). Is your own face more than a highly familiar face? Biological Psychology, 142, 100–107. 10.1016/j.biopsycho.2019.01.018 30738092

[bibr3-20416695221111409] Barton J. J. Radcliffe N. Cherkasova M. V. Edelman J. Intriligator J. M. (2006). Information processing during face recognition: The effects of familiarity, inversion, and morphing on scanning fixations. Perception, 35(8), 1089–1105. 10.1068/p5547 17076068

[bibr4-20416695221111409] Brady N. Campbell M. Flaherty M. (2005). Perceptual asymmetries are preserved in memory for highly familiar faces of self and friend. Brain and Cognition, 58(3), 334–342. 10.1016/j.bandc.2005.01.001 15963384

[bibr5-20416695221111409] Brédart S. (2003). Recognising the usual orientation of one’s own face: The role of asymmetrically located details. Perception, 32(7), 805–811. 10.1068/p3354 12974566

[bibr7-20416695221111409] Bruce V. (1982). Changing faces: Visual and non-visual coding processes in face recognition. British Journal of Psychology, 73(1), 105–116. 10.1111/j.2044-8295.1982.tb01795.x 7059746

[bibr8-20416695221111409] Buttle H. Raymond J. E. (2003). High familiarity enhances visual change detection for face stimuli. Perception & Psychophysics, 65(8), 1296–1306. 10.3758/BF03194853 14710963

[bibr9-20416695221111409] Caharel S. Fiori N. Bernard C. Lalonde R. Rebaï M. (2006). The effects of inversion and eye displacements of familiar and unknown faces on early and late-stage ERPs. International Journal of Psychophysiology, 62(1), 141–151. 10.1016/j.ijpsycho.2006.03.002 16678927

[bibr10-20416695221111409] Carey S. (1992). Becoming a face expert. Philosophical Transactions of the Royal Society of London. Series B: Biological Sciences, 335(1273), 95–103. 10.1098/rstb.1992.0012 1348143

[bibr11-20416695221111409] Davidoff J. Donnelly N. (1990). Object superiority: A comparison of complete and part probes. Acta Psychologica, 73(3), 225–243. 10.1016/0001-6918(90)90024-A 2353588

[bibr12-20416695221111409] DeGutis J. Cohan S. Mercado R. J. Wilmer J. Nakayama K. (2012). Holistic processing of the mouth but not the eyes in developmental prosopagnosia. Cognitive Neuropsychology, 29(5–6), 419–446. 10.1080/02643294.2012.754745 23428080

[bibr13-20416695221111409] Devue C. Brédart S. (2011). The neural correlates of visual self-recognition. Consciousness and Cognition, 20(1), 40–51. 10.1016/j.concog.2010.09.007 20880722

[bibr15-20416695221111409] Estudillo A. J. (2012). Facial memory: The role of the pre-existing knowledge in face processing and recognition. Europe’s Journal of Psychology, 8(2), 231–244. 10.5964/ejop.v8i2.455

[bibr16-20416695221111409] Estudillo A. J. (2017). Commentary: My face or yours? Event-related potential correlates of self-face processing. Frontiers in Psychology, 8, e608. 10.3389/fpsyg.2017.00608 PMC5397524 28473790

[bibr17-20416695221111409] Estudillo A. J. Bindemann M. (2014). Generalization across view in face memory and face matching. i-Perception, 5(7), 589–601. 10.1068/i0669 25926967 PMC4411982

[bibr18-20416695221111409] Estudillo A. J. Bindemann M. (2017a). A multi-sensory system for self-face learning. In Bindemann M. Megreya A. M. (Eds.), Face processing: systems, disorders and cultural differences (pp. 241–254). Nova Science Publisher.

[bibr19-20416695221111409] Estudillo A. J. Bindemann M. (2017b). Can gaze-contingent mirror-feedback from unfamiliar faces alter self-recognition? The Quarterly Journal of Experimental Psychology, 70(5), 944–958. 10.1080/17470218.2016.1166253 26982263

[bibr20-20416695221111409] Estudillo A. J. Kaufmann J. M. Bindemann M. Schweinberger S. R. (2018). Multisensory stimulation modulates perceptual and post perceptual face representations: Evidence from event–related potentials. European Journal of Neuroscience, 48(5), 2259–2271. 10.1111/ejn.14112 30107052

[bibr21-20416695221111409] Faul F. Erdfelder E. Lang A. G. Buchner A. (2007). G* power 3: A flexible statistical power analysis program for the social, behavioral, and biomedical sciences. Behavior Research Methods, 39(2), 175–191. 10.3758/BF03193146 17695343

[bibr22-20416695221111409] Gauthier I. Curran T. Curby K. M. Collins D. (2003). Perceptual interference supports a non-modular account of face processing. Nature Neuroscience, 6(4), 428–432. 10.1038/nn1029 12627167

[bibr23-20416695221111409] Greenberg S. N. Goshen-Gottstein Y. (2009). Not all faces are processed equally: Evidence for featural rather than holistic processing of one’s own face in a face-imaging task. Journal of Experimental Psychology: Learning, Memory, and Cognition, 35(2), 499–508. 10.1037/a0014640 19271862

[bibr24-20416695221111409] Gregory R. L. (2001). Seeing oneself. Perception, 30(8), 903–904. 10.1068/p3008ed 11578076

[bibr25-20416695221111409] Grill-Spector K. Kanwisher N. (2005). Visual recognition: As soon as you know it is there, you know what it is. Psychological Science, 16(2), 152–160. 10.1111/j.0956-7976.2005.00796.x 15686582

[bibr26-20416695221111409] Hills P. J. (2018). Children process the self face using configural and featural encoding: Evidence from eye tracking. Cognitive Development, 48, 82–93. 10.1016/j.cogdev.2018.07.002

[bibr27-20416695221111409] Hills P. J. Cooper R. E. Pake J. M. (2013). First fixations in face processing: The more diagnostic they are the smaller the face- inversion effect. Acta Psychologia, 142(2), 211–219. 10.1016/j.actpsy.2012.11.013 23348201

[bibr28-20416695221111409] Jacques C. Rossion B. (2006). The speed of individual face categorization. Psychological Science, 17(6), 485–492. 10.1111/j.1467-9280.2006.01733.x 16771798

[bibr29-20416695221111409] Keenan J. P. Wheeler M. A. Gallup G. G. Pascual-Leone A. (2000). Self-recognition and the right prefrontal cortex. Trends in Cognitive Sciences, 4(9), 338–344. 10.1016/S1364-6613(00)01521-7 10962615

[bibr30-20416695221111409] Keyes H. (2012). Categorical perception effects for facial identity in robustly represented familiar and self-faces: The role of configural and featural information. The Quarterly Journal of Experimental Psychology, 65(4), 760–772. 10.1080/17470218.2011.636822 22248095

[bibr31-20416695221111409] Keyes H. Brady N. (2010). Self-face recognition is characterized by “bilateral gain” and by faster, more accurate performance which persists when faces are inverted. The Quarterly Journal of Experimental Psychology, 63(5), 840–847. 10.1080/17470211003611264 20198537

[bibr32-20416695221111409] Keyes H. Brady N. Reilly R. B. Foxe J. J. (2010). My face or yours? Event- related potential correlates of self-face processing. Brain and Cognition, 72(2), 244–254. 10.1016/j.bandc.2009.09.006 19854553

[bibr34-20416695221111409] Laeng B. Rouw R. (2001). Canonical views of faces and the cerebral hemispheres. Laterality, 6(3), 193–224. 10.1080/713754410 15513170

[bibr35-20416695221111409] Leder H. Carbon C. C. (2005). When context hinders! learn–test compatibility in face recognition. The Quarterly Journal of Experimental Psychology Section A, 58(2), 235–250. 10.1080/02724980343000936 15903116

[bibr36-20416695221111409] Macchi Cassia V. Picozzi M. Kuefner D. Bricolo E. Turati C. (2009). Holistic processing for faces and cars in preschool–aged children and adults: Evidence from the composite effect. Developmental Science, 12(2), 236–248. 10.1111/j.1467-7687.2008.00765.x 19143797

[bibr37-20416695221111409] Maurer D. Le Grand R. Mondloch C. J. (2002). The many faces of configural processing. Trends in Cognitive Sciences, 6(6), 255–260. 10.1016/S1364-6613(02)01903-4 12039607

[bibr38-20416695221111409] McKone E. (2009). Holistic processing for faces operates over a wide range of sizes but is strongest at identification rather than conversational distances. Vision Research, 49(2), 268–283. 10.1016/j.visres.2008.10.020 19022276

[bibr39-20416695221111409] McKone E. Yovel G. (2009). Why does picture-plane inversion sometimes dissociate perception of features and spacing in faces, and sometimes not? Toward a new theory of holistic processing. Psychonomic Bulletin & Review, 16(5), 778–797. 10.3758/PBR.16.5.778 19815781

[bibr40-20416695221111409] Piepers D. Robbins R. (2012). A review and clarification of the terms “holistic”, “configural”, and “relational” in the face perception literature. Frontiers in Psychology, 3, e559. 10.3389/fpsyg.2012.00559 PMC3571734 23413184

[bibr41-20416695221111409] Platek S. M. Keenan J. P. Gallup G. G. Mohamed F. B. (2004). Where am I? The neurological correlates of self and other. Cognitive Brain Research, 19(2), 114–122. 10.1016/j.cogbrainres.2003.11.014 15019708

[bibr42-20416695221111409] Retter T. L. Rossion B. (2015). Global shape information increases but color information decreases the composite face effect. Perception, 44(5), 511–528. 10.1068/p7826 26422900

[bibr43-20416695221111409] Rezlescu C. Susilo T. Wilmer J. B. Caramazza A. (2017). The inversion, part-whole, and composite effects reflect distinct perceptual mechanisms with varied relationships to face recognition. Journal of Experimental Psychology: Human Perception and Performance, 43(12), 1961–1973. 10.1037/xhp0000400 28406690

[bibr414-20416695221111409] Richler, J. J., Mack, M. L., Palmeri, T. J., & Gauthier, I. (2011). Inverted faces are (eventually) processed holistically. *Vision Research, 51*(3), 333-342. 10.1016/j.visres.2010.11.014 21130798

[bibr44-20416695221111409] Richler J. J. Gauthier I. (2014). A meta-analysis and review of holistic face processing. Psychological Bulletin, 140(5), 1281–1302. 10.1037/a0037004 24956123 PMC4152424

[bibr45-20416695221111409] Richler J. J. Palmeri T. J. Gauthier I. (2012). Meanings, mechanisms, and measures of holistic processing. Frontiers in Psychology, 3, e553. 10.3389/fpsyg.2012.00553 PMC3520179 23248611

[bibr46-20416695221111409] Richler J. J. Tanaka J. W. Brown D. D. Gauthier I. (2008). Why does selective attention to parts fail in face processing? Journal of Experimental Psychology: Learning, Memory, and Cognition, 34(6), 1356–1368. 10.1037/a0013080 18980400

[bibr47-20416695221111409] Rossion B. (2008). Picture-plane inversion leads to qualitative changes of face perception. Acta Psychologica, 128(2), 274–289. 10.1016/j.actpsy.2008.02.003 18396260

[bibr48-20416695221111409] Rossion B. (2009). Distinguishing the cause and consequence of face inversion: The perceptual field hypothesis. Acta Psychologica, 132(3), 300–312. 10.1016/j.actpsy.2009.08.002 19747674

[bibr49-20416695221111409] Rossion B. (2013). The composite face illusion: A whole window into our understanding of holistic face perception. Visual Cognition, 21(2), 139–253. 10.1080/13506285.2013.772929

[bibr50-20416695221111409] Rossion B. Boremanse A. (2008). Nonlinear relationship between holistic processing of individual faces and picture-plane rotation: Evidence from the face composite illusion. Journal of Vision, 8(4), e3. 10.1167/8.4.3 18484842

[bibr51-20416695221111409] Rossion B. Gauthier I. (2002). How does the brain process upright and inverted faces? Behavioral and Cognitive Neuroscience Reviews, 1(1), 63–75. 10.117/1534582302001001004 17715586

[bibr52-20416695221111409] Rossion B. Retter T. L. (2015). Holistic face perception: Mind the gap! Visual Cognition, 23(3), 379–398. 10.1080/13506285.2014.1001472

[bibr53-20416695221111409] Sekuler A. B. Gaspar C. M. Gold J. M. Bennett P. J. (2004). Inversion leads to quantitative, not qualitative, changes in face processing. Current Biology, 14(5), 391–396. 10.1016/j.cub.2004.02.028 15028214

[bibr54-20416695221111409] Susilo T. Rezlescu C. Duchaine B. (2013). The composite effect for inverted faces is reliable at large sample sizes and requires the basic face configuration. Journal of Vision, 13(13), e14. 10.1167/13.13.14 24222184

[bibr55-20416695221111409] Tanaka J. W. Curran T. Porterfield A. L. Collins D. (2006). Activation of preexisting and acquired face representations: The N250 event-related potential as an index of face familiarity. Journal of Cognitive Neuroscience, 18(9), 1488–1497. 10.1162/jocn.2006.18.9.1488 16989550

[bibr56-20416695221111409] Tanaka J. W. Farah M. J. (1993). Parts and wholes in face recognition. The Quarterly Journal of Experimental Psychology, 46(2), 225–245. 10.1080/1464074930840104 8316637

[bibr57-20416695221111409] Tanaka J. W. Gordon I. (2011). Features, configuration and holistic face processing. In Calder A. J. Rhodes G. Johnson M. H. Haxby J. V. (Eds.), The Oxford handbook of face perception (pp. 15–30). Oxford University Press.

[bibr58-20416695221111409] Tanaka J. W. Simonyi D. (2016). The “parts and wholes” of face recognition: A review of the literature. The Quarterly Journal of Experimental Psychology, 69(10), 1876–1889. 10.1080/17470218.2016.1146780 26886495 PMC5051945

[bibr59-20416695221111409] Tong F. Nakayama K. (1999). Robust representations for faces: Evidence from visual search. Journal of Experimental Psychology: Human Perception and Performance, 25(4), 1016–1035. 10.1037/0096-1523.25.4.1016 10464943

[bibr60-20416695221111409] Troje N. F. Kersten D. (1999). Dependent recognition of familiar faces. Perception, 28(4), 483–487. 10.1068/p2901 10664788

[bibr61-20416695221111409] Van Belle G. De Graef P. Verfaillie K. Busigny T. Rossion B. (2010a). Whole not hole: Expert face recognition requires holistic perception. Neuropsychologia, 48(9), 2620–2629. 10.1016/j.neuropsychologia.2010.04.034 20457169

[bibr62-20416695221111409] Van Belle G. De Graef P. Verfaillie K. Rossion B. Lefèvre P. (2010b). Face inversion impairs holistic perception: Evidence from gaze-contingent stimulation. Journal of Vision, 10(5), e10. 10.1167/10.5.10 20616142

[bibr63-20416695221111409] Veres-Injac B. Persike M. (2009). Recognition of briefly presented familiar and unfamiliar faces. Psihologija, 42(1), 47–66. 10.2298/PSI0901047V

[bibr64-20416695221111409] Willenbockel V. Fiset D. Chauvin A. Blais C. Arguin M. Tanaka J. W. Bub D. N. Gosselin F. (2010). Does face inversion change spatial frequency tuning? Journal of Experimental Psychology: Human Perception and Performance, 36(1), 122–135. 10.1037/a0016465 20121299

[bibr65-20416695221111409] Wong A. C. N. Palmeri T. J. Gauthier I. (2009). Conditions for facelike expertise with objects: Becoming a ziggerin expert—but which type? Psychological Science, 20(9), 1108–1117. 10.1111/j.1467-9280.2009.02430.x 19694980 PMC2919853

[bibr66-20416695221111409] Xu B. Tanaka J. W. (2013). Does face inversion qualitatively change face processing: An eye movement study using a face change detection task. Journal of Vision, 13(2), e22. 10.1167/13.2.22 23420421

[bibr67-20416695221111409] Yin R. K. (1969). Looking at upside-down faces. Journal of Experimental Psychology, 81(1), 141–145. 10.1037/h0027474

[bibr68-20416695221111409] Young A. W. Burton A. M. (2018). Are we face experts? Trends in Cognitive Sciences, 22(2), 100–110. 10.1016/j.tics.2017.11.007 29254899

[bibr69-20416695221111409] Young, A. W., Hay, D. C., McWeeny, K. H., Flude, B. M., & Ellis, A. W. (1985). Matching familiar and unfamiliar faces on internal and external features. *Perception, 14*(6), 737–746. 10.1068/p140737 3837875

